# Is Nephrolithiasis an Unrecognized Extra-Articular Manifestation in Ankylosing Spondylitis? A Prospective Population-Based Swedish National Cohort Study with Matched General Population Comparator Subjects

**DOI:** 10.1371/journal.pone.0113602

**Published:** 2014-11-25

**Authors:** Ane Krag Jakobsen, Lennart T. H. Jacobsson, Oliver Patschan, Johan Askling, Lars Erik Kristensen

**Affiliations:** 1 Department of Urology, Skåne University Hospital, Malmö, Sweden; 2 Department of Rheumatology and Inflammation Research, Sahlgrenska Academy at University of Gothenburg, Gothenburg, Sweden; 3 Clinical Epidemiology Unit and Rheumatology Unit, Department of Medicine (Solna), Karolinska Institutet, Stockholm, Sweden; 4 The Parker Institute and Department of Rheumatology; Frederiksberg and Bispebjerg, Denmark; 5 Department of Rheumatology, Lund University, Malmö, Sweden; University of Texas Health Science Center at Houston, United States of America

## Abstract

**Background:**

Ankylosing spondylitis (AS) is associated with several extra-articular manifestations. Nephrolithiasis (NL) has not been recognized as one of those, however, several factors known to increase the risk of NL are at play in AS patients. The objective was to estimate rates and predictors of NL in Swedish patients with AS compared to the general population.

**Methods and Findings:**

We performed a prospective population-based nationwide cohort study based on linkage of data from Swedish registries. 8,572 AS patients were followed for 49,258 person-years (py) and 39,639 matched general population comparators were followed for 223,985 py. Patients were followed prospectively together with comparator subjects from January 2001 through December 2009. The first occurrence of NL during follow-up was the primary outcome. Hazard Ratios (HR) were used to compare these rates adjusting for comorbidities and treatment, and to assess predictors for NL. Mean age at study entry was 46 years (inter quartile range 36–56 years), 65% were males. Based on 250 vs. 466 NL events, the adjusted HR of NL in AS patients was 2.1 (95%CI 1.8 to 2.4). Predictors of NL within the AS group included prior diagnosis of inflammatory bowel disease (IBD) (HR 2.3; 95%CI 1.7 to 3.3), prior diagnosis of NL (HR 16.4; 95%CI 11.5 to 23.4), and patients receiving anti-TNF treatment (HR 1.6; 95%CI 1.2 to 2.1). Male sex was a risk factor for NL both in AS patients and in the general population.

**Limitations:**

The risk for residual confounding and inability to study the chemical nature of NL were considered the main limitations of the study.

**Conclusions:**

Patients with AS are at increased risk of NL, which may be considered a novel extra-articular manifestation. Previous history of NL, IBD, AS disease severity and male sex were identified as predictors of NL in AS.

## Introduction

Kidney stone formation is recognized as a multifactorial condition with a diversity of underlying disorders and complex pathophysiological mechanisms involving several other organ systems besides the kidney [1], [2]. The yearly incidence of nephrolithiasis (NL) is estimated to be about 0.5–1.0%, with a lifetime risk of 10–25% and a recurrence rate of 50% in 5–10 years [3]–[6]. NL is associated with significant socioeconomic expenses due to medical costs and time lost from work [7].

The basic process of stone formation in the kidney is explained by supersaturation in the urine of organic and inorganic solutes, leading to precipitation and thus stone formation. In addition to supersaturation, urinary pH and concentration of inhibitory factors, such as magnesium and citrate, are involved in the process of lithogenesis [1], [2], [8].

Calcium containing stones constitute about 85% of all NL, while uric acid stones represent about 5–10%. The remainder are comprised of cystine stones, infection stones and other rare forms of NL [1], [2]. Drug-induced stone formation is negligible with an estimated frequency of 0.5–2% of all NL [9], [10], [11].

Approximately 80% of calcium containing stones are made up of calcium-oxalate, while 15% are composed of calcium-phosphate [1], [2]. Abnormalities known to be at play in some, but not all calcium stone forming individuals, include low urinary volume, disturbances in pH, hypercalciuria, hyperoxaluria, hyperuricosuria and hypocitraturia. These conditions may in turn be the consequence of a wide variety of disorders causing disturbances in intestinal absorption, renal resorption and skeletal remodelling [1].

Ankylosing Spondylitis (AS) is a chronic inflammatory joint disease with primary involvement of the spine and sacroiliac joints causing back pain and progressive stiffness of the spine. In addition, AS is characterized by potential musculoskeletal manifestations including peripheral arthritis and enthesitis as well as extra-articular manifestations, such as uveitis, inflammatory bowel disease (IBD), renal disease and psoriasis [12].

The prevalence of AS in the Scandinavian countries is estimated to be 0.1–0.2% with an annual incidence of around 7 per 100,000 [13], [14].

Several of the afore-mentioned predisposing factors observed in NL may also be present in AS patients, namely disturbances in intestinal absorption owing to clinical (10–15% of AS) and subclinical (up to 60% of AS) bowel inflammation [15]–[19], defective renal resorption due to renal disease [20]–[22], altered calcium metabolism due to inflammatory cytokines [23], [24] and spinal immobility causing altered bone-remodelling [24]–[26].

The aim of this study was therefore to compare the occurrence of NL in the general population and in AS patients in Sweden. Furthermore we assessed predictors of NL including comorbidities and AS disease severity. To do this, we used a population-based prospective cohort design with linkage of several national population- and healthcare registers.

## Methods

This is a Swedish population- and register-based nationwide prospective cohort study using register data from 1997 through December 31^st^, 2009 comparing AS patients to matched general population subjects.

Ethical approval for the study was granted by the Regional Ethics Committee, Karolinska Institutet, Stockholm, Sweden (ethical number: 2011/29-31/1). No informed consent was applicable as the study only involved quality register linkage, and no actual handling of patients. The ethics committee approved this consent procedure.

### Data Sources

On December 31st 2009, Sweden had a population of approximately 9.2 million.

Health- and demographic information on all inhabitants is updated annually in a series of national registers, with a very high degree of completeness [27]. Linkage of data from these registers is possible using the 10-digit personal identification number automatically assigned to all Swedish residents.

The Swedish healthcare system is tax funded and offers universal access. Data on health care contacts at inpatient (somatic: starting 1964, psychiatric: starting 1973) and non-primary outpatient (starting 2001) facilities are registered in the *Swedish Patient Register*, including date of contact and diagnoses given by the treating physician according to the Swedish version of the International Statistical Classification of Diseases (ICD-10 starting 1997). Reporting of data on each single health care contact, excluding primary care visits, is statutory.

The vast majority of patients with AS are diagnosed by rheumatologists at public outpatient and inpatient facilities. Patients with NL are also diagnosed and treated both in the inpatient and outpatient setting, but by physicians from a wider variety of specialities including urologist, general surgeons, specialists in acute medicine, specialists in internal medicine and general practitioners.

### Study population

We identified a prospective national population-based cohort of AS patients, using data from *The Swedish Patient Register*. Patients 16 years or older, who attended an outpatient clinic (Rheumatology or Internal Medicine Dept.) during the time period Jan 1^st^ 2001 through Dec 31^st^ 2009 and who received at least one ICD-coded diagnosis corresponding to AS (i.e. ICD10: M459) were included. Patients with a previously or concomitantly registered diagnosis code of systemic lupus erythematosus (SLE; ICD-10: M32.0, M32.1, M32.8, M32.9) or juvenile inflammatory arthritis (JIA; ICD-10: M08-09)) were excluded from the analyses.

A separate validation study currently in peer review revealed a validity of more than 90% of the AS diagnosis in this cohort.

Through register linkage, data on death, emigration and level of education (< = 9 years, 10 to 12 years, >12 years) were retrieved from *the Swedish Population Register, the Swedish Cause of Death Register and the Swedish Register of Education*.

For each AS patient up to 5 general population comparators, alive and without AS by the time of the index patients' first AS diagnosis during the study period were identified (from *the Swedish Population Register)* and matched on year of birth, sex and county.

### Follow-up

**Figure 1:**
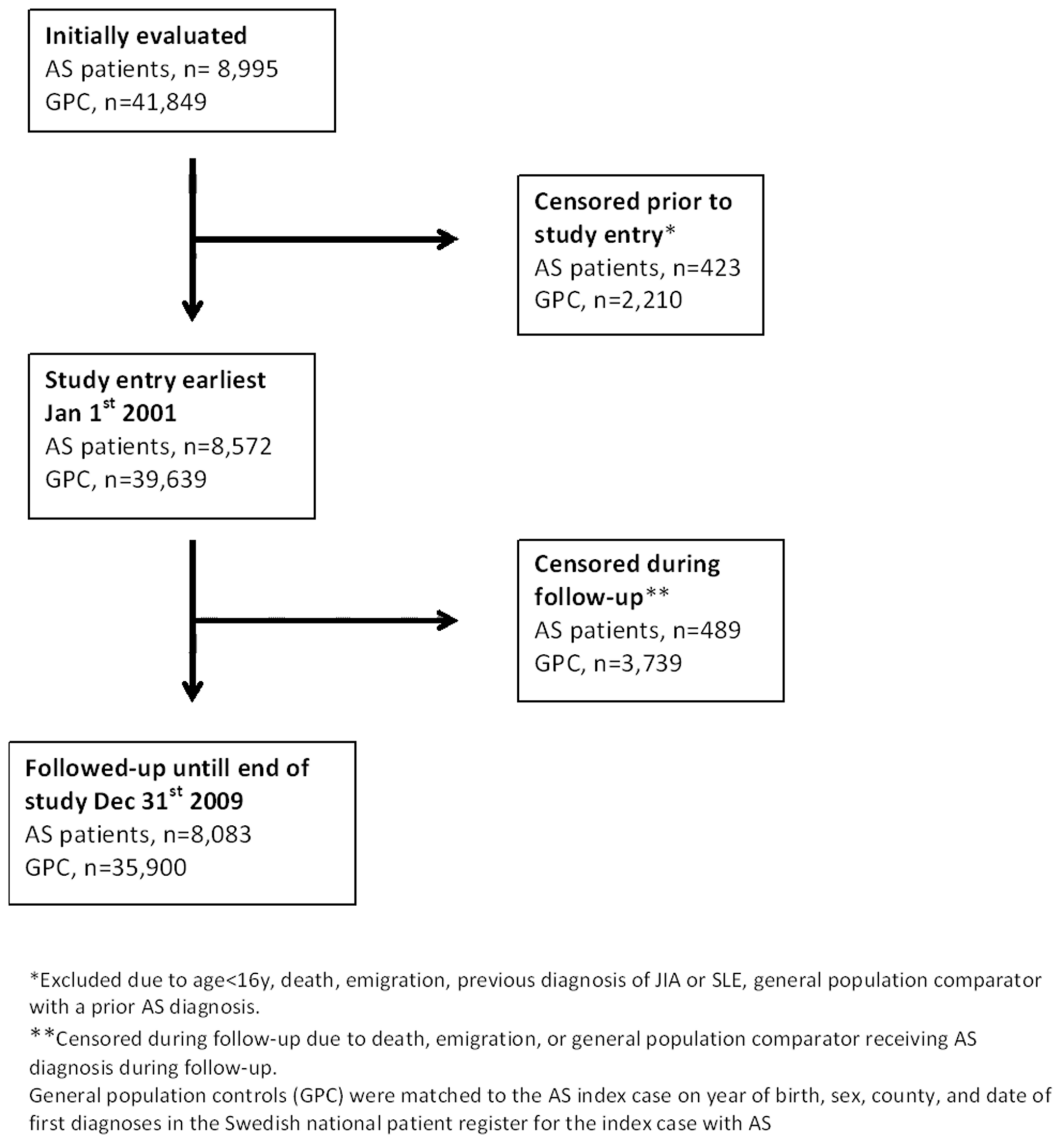
Flow of AS patients and general population comparators (GPC) prior to and during follow-up, until end of study period.

Cases and matched general population comparator subjects contributed to ‘time-at-risk’ from the time of study entry (Jan 1^st^ 2001 for those registered with AS diagnosis prior to or at that date, and from the date of AS diagnosis for those with their first AS diagnosis after Jan 1^st^ 2001) until December 31^st^ 2009, death, emigration or first NL diagnosis during follow-up, whichever came first. **Figure 1** illustrates inclusion, exclusion, censoring and losses to follow up of AS patients and general population comparators prior to and during the study period. Patients with a registered AS diagnosis prior to start of follow-up, were defined as “prevalent AS”, whereas patients receiving their first AS diagnosis after start of follow-up, were defined as “incident AS”.

### Outcome and potential predictors and confounders

Data on NL diagnosis (ICD-10: N20, N23.9) registered by physicians in hospital-based inpatient or outpatient somatic care clinics in AS patients and general population comparator subjects, prior to study entry (January 1^st^ 1997 through December 31^st^ 2000) and during follow-up (Jan 1^st^ 2001 thru Dec 31^st^ 2009), were retrieved from *The Swedish Patient Register*.

Our primary outcome was defined as the first registered main diagnosis of NL during follow-up, regardless of any NL diagnosis prior to follow-up.

Data on NL diagnosis and other clinically relevant comorbidities (see table S1) registered prior to study entry (from *The Swedish Patient Register*) and data on treatment with TNF inhibitors prior to study entry in AS patients (*The Swedish Biologics Register*) was used to describe baseline characteristics and to adjust for confounding and identify potential predictors.

### Statistical analysis

Crude incidence rates (IRs) of NL diagnosis per 1,000 person-years (py) with 95% confidence intervals were calculated for the AS patients and the matched general population comparator subjects, overall and stratified by sex. A multivariate Cox regression model adjusting for potential confounding was used to calculate hazard ratios and 95% confidence intervals (HR; 95%CI). Variables tested were chosen based on á priori clinical important factors including comorbidities and extra-articular manifestations, i.e. a history at start of follow-up (yes/no) of the following: atherosclerotic heart disease, hypertension, renal insufficiency, calcium metabolic disorders (hyperparathyroidism, hypervitaminosis D, osteoporosis, renal osteodystrophy, sarcoidosis), diabetes and other endocrine disorders, nephrolithiasis, uric acid disorders, cystinuria, hyperoxaluria, psoriasis, IBD, uveitis, urethritis (for definitions and ICD-codes used see Table S1), and age (per year). Moreover, treatment with TNF inhibitors prior to or during the study period was used as time-dependent binary variable. The assumptions for using Cox regression analyses were tested and found valid.

Based on backward deletion (p>0.15) the following variables were entered in the multivariate regression model: age, prior diagnosis of NL, prior diagnosis of IBD and use of anti-TNF treatment. Patients with AS were compared to general population comparator subjects in a univariate Cox regression model as well as a multivariate model adjusting for the identified confounders. All Cox regression models were stratified for sex.

Sensitivity analyses studying subsets of AS patients and corresponding controls were performed regarding prevalent and incident AS patients, excluding subjects with observation less than a year, studying the subset of subjects with recorded inpatient or outpatient visits for other reasons than AS or NL and excluding patients with NL prior to start of follow-up.

## Results

### Baseline characteristics

A total of 8,572 AS patients and 39,639 matched general population comparator subjects were included in the study and contributed 49,258 and 223,985 person-years, respectively. (py).

Mean age of AS patients and general population comparator subjects at study entry was 46 years (inter quartile range 36–56 years), 65% were male. Since some AS patients in our unselected study population received their diagnosis years prior to study entry, the mean age is higher than what would be expected from an incident AS population. Baseline characteristics of AS patients and general population comparator subjects are presented in **table 1**. Baseline data were complete except for data on education level, which was missing in 1.2% (general population n = 457, AS n = 101). The baseline data on demographics and extra-articular comorbidities for the AS group resembles a population of moderate to severe AS. As would be expected, AS patients and general population comparators differed at study entry regarding history of AS-related comorbidities. In addition, AS patients had a higher frequency of prior NL diagnosis, whereas frequency of obesity and diabetes was similar in the two groups.

**Table 1:** Baseline characteristics of AS cohort and matched general population comparator subjects at study entry.

		AS cohort	General population controls
		n = 8,572	n = 39,639
**Male, % (n)**		65.3 (5,597)	65.0 (25,769)
**Age at study entry, mean (interquartile range)**		46.4 (36.0–56.0)	46.1 (36.0–56.0)
**Education, % (n)**	≤9 y	21.7 (1,859)	21.3 (8,436)
	10–12 y	47.2 (4,042)	46.6 (18,474)
	≥12 y	30.0 (2,570)	31.0 (12,272)
	Missing	1.2 (101)	1.2 (457)
**Medication, % (n)**	Anti-TNF	8.5 (730)	Na
**Comorbidities, % (n)**	Uveitis	11.4 (978)	0.2 (93)
	Renal insufficiency	7.5 (644)	3.6 (1,428)
	Hypertension	7.3 (626)	3.5 (1,389)
	IBD	6.0 (515)	0.8 (303)
	Ischemic heart disease	5.1 (434)	3.2 (1,276)
	Diabetes	3.9 (337)	3.2 (1,267)
	Psoriasis	3.2 (271)	0.5 (211)
	Nephrolithiasis	1.6 (134)	0.7 (273)
	Calcium metabolic disorders	1.2 (100)	0.4 (154)
	Arthritis urica, cystinuria, hyperoxaluria	0.4 (31)	0.1 (38)
	Obesitas	0.4 (37)	0.4 (173)

### Nephrolithiasis risk in AS patients vs general population comparators

Table 2 summarises the number of NL events, time at risk, and crude IRs of NL occurring in AS patients and general population comparators during the study period. A total of 466 NL events (max one per person during follow-up) were recorded in the overall general population cohort during the study period, corresponding to a crude IR per 1,000 py of 2.1 (95%CI 1.9 to 2.3). By contrast, 250 NL events were recorded in the AS cohort, corresponding to a crude IR of 5.1 per 1,000 py (95%CI 4.5 to 5.8). The unadjusted HR of NL in AS compared to population comparators was 2.4 (95%CI 2.1 to 2.9).

**Table 2:** Number of events and Incidence rates of first NL diagnosis during study period in AS patients (n = 8,572) compared to general population comparators (n = 39,639), overall and stratified by status on NL diagnosis prior to study entry.

		No. of NL	Time at risk, years	Crude IR per 1,000 py (95%CI)
		events/py	Median (IQR)	
Overall	GPC	466/223,985	6.1 (3.1 to 8.6)	2.1 (1.9 to 2.3)
Overall	AS	250/49,258	6.3 (3.2 to 8.7)	5.1 (4.5 to 5.8)
No prior NL	GPC	429/222,995	6.2 (3.1 to 8.6)	1.9 (1.8 to 2.1)
No prior NL	AS	212/48,806	6.3 (3.3 to 8.7)	4.3 (3.8 to 5.0)
Prior NL	GPC	37/990	3.1 (1.3 to 5.7)	37.4 (26.3 to 51.5)
Prior NL	AS	38/452	2.7 (1.0 to 5.3)	84.1 (59.5 to 115.4)

GPC; general population comparators.

py; person-years. Person years defined as starting at the date of entry into study (time of AS diagnosis, earliest Jan 1^st^ 2001) until first of death, emigration, nephrolithiasis diagnosis or end of study (Dec 31^st^ 2009).

IQR; interquartile range, IR; incidence rate, CI; confidence interval.

**Figure 2:**
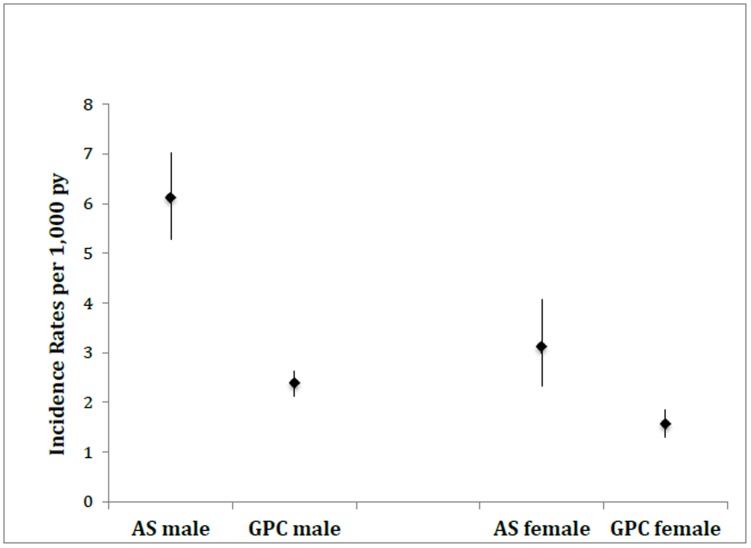
Incidence Rates (95%CI) of NL diagnosis in AS patients and general population comparators (GPC).

As illustrated in **figure 2** the increased risk of NL diagnosis in AS patients was evident in both sexes compared to the matched subjects with a rate ratio of 2.6 (95%CI 2.0 to 2.4) for males and 2.0 (95%CI 1.4 to 2.8) for females.

A stratified univariate analyses according to NL diagnosis prior to study entry were performed for AS patients compared to general population comparators. This revealed a HR of 2.3 (95%CI 1.9 to 2.7) in AS patients without prior NL diagnosis at start of follow-up, and similar HR (2.2, 95%CI 1.4 to 3.5) in AS patients with a history of NL diagnosis.

At the end of the study period 4.0% of the AS patients and 1.8% of the general population comparators had received a NL diagnosis, during and/or prior to start of follow-up.

### Confounders and predictors of nephrolithiasis in AS patients

Among AS patients anti-TNF treatment (HR 1.6, 95% CI 1.2 to 2.1), IBD (2.3 (95%CI 1.7 to 3.3), and a previous history of NL (16.4 (95%CI 11.5 to 23.4)) were identified as predictors of NL during follow-up (**table 3**).

**Table 3:** HRs (95%CI) for all covariates included in initial multivariate Cox regression model.

Covariates	HR (95%CI)	
**Age at study entry (years)**	1.01 (1.00 to 1.02)	*p* = 0.107
**Anti-TNF (yes/no)**	1.50 (1.13 to 2.05)	*p* = 0.006
**Comorbidities (yes/no)**		
**Uveitis**	0.86 (0.55 to 1.34)	*p* = 0.500
**Renal insufficiency**	1.57 (0.22 to 11.24)	*p* = 0.656
**Hypertension**	0.64 (0.09 to 4.80)	*p* = 0.665
**IBD**	2.28 (1.60 to 3.25)	*p*<0.001
**Ischemic heart disease**	0.81 (0.46 to 1.44)	*p* = 0.479
**Diabetes**	1.08 (0.53 to 2.19)	*p* = 0.831
**Psoriasis**	0.45 (0.14 to 1.42)	*p* = 0.172
**Nephrolithiasis**	16.02 (11.03 to 23.27)	*p<0.001*
**Calcium metabolic disorders**	0.97 (0.39 to 2.41)	*p = 0.945*
**Arthritis urica, cystinuria, hyperoxaluria**	1.58 (0.22 to 11.46)	*p* = 0.654
**Obesitas**	1.75 (0.42 to 7.34)	*p* = 0.441

When comparing AS patients to general population subjects with adjustment for age, IBD, and prior NL, AS remained a highly significant risk factor with a HR of 2.1 (95%CI 1.8 to 2.4).

Moreover an even higher risk of NL was found in AS patients treated with anti-TNF with a multivariate HR of 3.0 (95% CI 2.3 to 3.9) compared to the general population group. When excluding patients and matched subjects with the identified predictors from the analysis, the remaining AS patients had an increased risk of 2.0 (95% CI 1.6 to 2.4).

### Sensitivity analysis

In order to control for selection bias and detection bias, various sensitivity analysis were performed. A subgroup analysis showed a persistently increased risk of NL in AS compared to general population subjects in both prevalent AS (HR 3.6, 95% CI 2.8 to 4.7) and incident AS (HR 2.0, 95% CI 1.6 to 2.4). When excluding patients with prior NL, the risk of de novo NL remained increased for incident AS patients (HR 1.8, 95% CI 1.4 to 2.4).When excluding AS patients and comparator subjects with observation time less than a year (due to death, emigration or NL diagnosis during the first year after inclusion), AS patients still had an increased risk for NL compared to general population (HR 2.4, 95% CI 2.0 to 2.8). Moreover, when studying the subgroup of patients and comparators having attended hospital care prior to study entry for other common reasons not associated with NL (hypertension, diabetes, ischemic heart disease or renal disease), AS patients remained at increased risks for NL (HR 1.6, 95% CI 1.1 to 2.5).

## Discussion

This study demonstrates a more than two-fold increased risk of kidney stone diagnosis in AS patients compared to the general population, confirming our prespecified hypothesis.

Male sex, prior diagnosis of IBD, prior diagnosis of NL, and patients receiving anti-TNF therapy were identified as predictors of NL in AS patients. These findings are consistent with previously established risk factors of NL [3]–[6], [28]. Notably, the risk of NL in AS patients remained more than two-fold increased after adjusting for these confounders. This suggests that AS in itself or through other closely related factors may contribute to the process of kidney stone formation. Comorbidities such as renal insufficiency and calcium metabolic disorders did not significantly predict risk of NL.

The crude incidence rate of NL in general population subjects of 2.1/1,000 py (95%CI 1.9 to 2.3) found in this study, is comparable to incidence rates reported in other studies on populations in industrialized countries [3]–[6] which supports the validity of our findings.

In this study we used treatment with TNF-inhibitors as a surrogate marker of more severe AS. The fact that patients receiving TNF-inhibitors had a 1.6-fold increased risk of NL compared to other AS patients and 3-fold increased risk compared to general population subjects, suggests that disease severity may be associated with a greater risk for NL. However less likely, we cannot rule out the possibility that TNF-inhibitor treatment in itself increases the risk of NL. Consistently, the various sensitivity analyses supported the increased risk of NL in AS patients. Importantly, incident AS patients without any prior history of NL at baseline had a 1.8 fold increased risk of NL compared to matched comparator subjects. This supports a causative association between AS and NL.

To our knowledge this is the first study to assess the risk of NL diagnosis in AS patients compared to matched general population subjects based on nationwide prospective data. This association has previously been suggested by a few studies. However these studies were based on cross-sectional data in selected AS populations diagnosed and treated at single outpatient centre. Sample size was small (between 53 and 163 AS patients). One study lacked control subjects, while the other 2 included small control groups selected in the same setting and without any matching [29]–[31].

The Swedish National Patient Register consisting of the Inpatient Register (starting 1964) and the Outpatient Register (starting 1997) is a substantial data source in this study. All physicians in the country working in both publicly funded as well as private (2001) healthcare units are obliged to report data, including personal identity number and ICD-coded diagnosis, on all in-patient and specialist out-patient visits.

Evaluations of data in the Inpatient Register have shown validity between 85–95% across different diagnoses and coverage of more than 99% [32], [33]. Regarding data on specialist outpatient visits, the overall coverage of 80% is somewhat lower. This is primarily explained by missing data from private caregivers, whereas coverage from public non primary care outpatient units is almost 100% [33].

Thus nationwide register-based studies like the present have the apparent strength of being population-based reducing the risk of selection bias. In addition, the large sample size allows for adjustment and sensitivity analyses [34]. However some degree of residual confounding and bias cannot be ruled out.

Selection of AS patients in this study is based on ICD-codes recorded by a specialist in rheumatology or internal medicine. This might create a selection bias towards more severe cases being included, while missing patients with mild disease who are managed entirely at primary care units. However, according to a previous study in the same setting, this is a minor problem and would only increase the number of cases by less than 4%, at the expense of a larger degree of misclassification [35]. Regarding the case definitions of AS used in this study unpublished data and results from southern Sweden suggest that misclassification occurs in less than 10% [36].

Concerning comorbidities such as acute coronary events, misclassification is estimated to be less than 5% [32], [37].

Data on NL as well as comorbidities in this study is based on ICD-codes recorded by physicians at inpatient and outpatient somatic care units irrespective of speciality. Thus our results do not account for patients who are exclusively diagnosed and treated in primary care units. This may be the case for some of the relevant comorbidities such as obesity, DM, hyperlipidaemia and hypertension. In addition some relevant comorbidities such as calcium metabolic disorders and renal disease may remain undiagnosed/subclinical.

Regarding NL diagnosis we assume that a high proportion of all patients with symptomatic and thus significant NL are managed in inpatient or outpatient specialist care units and thus have a recorded diagnosis of NL. However we cannot rule out the possibility, that a proportion of patients with asymptomatic or mild NL passing spontaneously are diagnosed and treated entirely in primary care.

Thus, our data could have been affected by differential ascertainment of both outcome and comorbidities owing to more frequent contact to health care professionals in the AS population. In order to examine the impact of this potential detection bias, sensitivity analyses were performed. When excluding the first year of observation after study entry the risk for NL remained increased. This risk increment was also notable for prevalent and incident AS cases analyzed separately. In addition, a subgroup analysis of patients with prior common comorbidities also showed an increased risk of NL in AS patients. This supports that attendance to hospital care and elicited investigations associated with a recent AS diagnosis, did not explain the increased risk for NL. Together, the sensitivity analyses support that AS in itself is associated with an increased risk for NL.

## Interpretation- Generalizability

Based on these findings of a more than two-fold increased risk compared to the general population, we suggest nephrolithiasis as a hitherto unrecognized extra-articular manifestation in AS-patients, which seems to be correlated to disease severity.

The fact that NL is present in about 1 of 20 AS patients, should be kept in mind by clinicians treating AS patients, especially in patients with concomitant IBD and those with a prior diagnosis of nephrolithiasis.

The increased risk of NL in AS patients is only partly explained by the higher prevalence of IBD. Several other mechanisms are also expected to be involved, consistent with the known multifactorial nature of kidney stone formation. Further studies are encouraged in order to clarify these mechanisms. Some of the questions to be answered are, what kind of stones and what kind of urinary abnormalities are found in AS patients? What role does disorders of the intestine, kidney, skeleton, immune system and other organ systems play?

## Conclusions

The risk of NL diagnosis in AS patients compared to the general population is more than two-fold increased and seems to correlate with disease severity. Based on these findings, NL is suggested as an extra-articular manifestation in AS. In addition male sex, prior IBD diagnosis and prior NL are significant and clinical important predictors of NL in AS patients.

## Supporting Information

Table S1 **List of diagnoses and corresponding ICD-10 codes.**
(DOCX)
