# Supplementary material for: Is Nephrolithiasis an Unrecognized Extra-Articular Manifestation in Ankylosing Spondylitis? A Prospective Population-Based Swedish National Cohort Study with Matched General Population Comparator Subjects
Source: PLoS One. 2014 Nov 25;9(11):e113602. doi: 10.1371/journal.pone.0113602 (PMC4244108; doi:10.1371/journal.pone.0113602)
Supplement: Table S1 — List of diagnoses and corresponding ICD-10 codes. (DOCX) [file pone.0113602.s001.docx]

| **Diagnosis** | **ICD-10** |
| --- | --- |
| AS | M45 (ICD-9: 720A; ICD-8: 712.40, 726.99) |
| Uveitis | H20, H221 |
| IBD | K50-K51 |
| Psoriasis | L40 |
| Renal insufficiency | E10.2C, E11.2C, I12.0, I12.9, I13.1-I13.2, N17.0-N17.9, N18.9, N19.9, N28.0, N99.0, O90.4 |
| Hypertension | I10-I15 |
| Obesitas | E66 |
| Diabetes | E10-E14, O24 |
| Ischemic heart disease | I20 – I25 |
| Nephrolithiasis | N20, N23.9 |
| Calcium metabolic disorders | E21, E67.3, M80, M81, M82, N25.0, D86 |
| Arthritis urica, cystinuria, hyperoxaluria | M10, E79, E74.8B, E72.0B |

**Table S1:** list of diagnoses and corresponding ICD-10 codes.
